# Treatment strategy optimization for patients with non-small-cell lung cancer harboring EGFR mutation: a Delphi consensus

**DOI:** 10.1007/s12094-020-02518-0

**Published:** 2020-11-18

**Authors:** D. Isla, J. de Castro, R. García-Campelo, M. Majem, D. Vicente, O. Juan-Vidal

**Affiliations:** 1 Medical Oncology Department, University Hospital Lozano Blesa, IIS Aragón, Avda. San Juan Bosco, 15, 50009 Zaragoza, Spain; 2 Medical Oncology Department, University Hospital La Paz-IDIPAZ, Madrid, Spain; 3 Medical Oncology Unit, University Hospital A Coruña, A Coruña, Spain; 4 Medical Oncology Unit, Hospital Santa Creu I Sant Pau, Barcelona, Spain; 5 Medical Oncology Unit, University Hospital Virgen Macarena, Sevilla, Spain; 6 Thoracic Oncology Unit, Department of Medical Oncology, Hospital Universitari I Politècnic La Fe, Av. Fernando Abril Martorell 106, 46026 Valencia, Spain

**Keywords:** Delphi, Non–small-cell lung cancer, Epidermal growth factor receptor, Tirosine kinase inhibitors

## Abstract

**Aim:**

To stablish a consensus on the treatment strategy for advanced non–small-cell lung cancer (aNSCLC) with epidermal growth factor receptor mutation (EGFRm) in Spain.

**Methods:**

After a systematic literature review, the scientific committee developed 33 statements in 4 fields: molecular diagnosis (10 items); histologic profile and patient clinical characteristics (7 items); first-line (1L) treatment in EGFRm aNSCLC (8 items); and subsequent-line treatment (8 items). A panel of 31 experts completed 2 Delphi online questionnaires rating their degree of agreement/disagreement for each statement through a 1–9 range scale (1–3 = disagree, 7–9 = agree). Consensus was reached if 2/3 of the participants are in the median range.

**Results:**

In the first Delphi round consensus was achieved for 24/33 of the statements. One of the assertions was deleted, proceeding to a second round with the eight remaining questions with no consensus or in the range of indeterminacy. Determination of the EGFR status from tissue and analysis of the different biomarkers are two important variables that influenced treatment decision in patients with aNSCLC. 1L treatment should be the best therapeutic option, independently of the subsequent lines of treatment. For patients with the most common activating mutations osimertinib was considered the most efficient and safe 1L option. In case of disease progression, a new biopsy was needed.

**Conclusions:**

A consensus document is proposed to optimize the treatment strategy for untreated patients with a NSCLC with EGFR sensitizing mutations.

## Introduction

Non-small cell lung cancer (NSCLC) account for 85%–90% of lung cancer, being adenocarcinoma the most common subtype. Epidermal growth factor receptor (EGFR) mutations are found in ∼10%–12% of Caucasians with adenocarcinoma and are more frequent in never smokers, females and in patients of East Asian ethnicity [1, 2]. EGFR mutation (EGFRm) testing is recommended in all patients with advanced non-squamous cell carcinoma, it is not recommended in patients with an unequivocal diagnosis of squamous cell carcinoma with the exception of never/former light smokers (< 15 pack years). The discovery of EGFRm and development of EGFR tyrosine kinase inhibitors (TKIs) have achieved a paradigm shift in treatment strategy of advanced NSCLC (aNSCLC). Several randomized phase III studies have revealed that treatment with first- or second-generation EGFR-TKIs results in an improved progression-free survival (PFS) compared to standard chemotherapy in treatment naïve patients with aNSCLC [3]. Results from direct comparison of first-, second-, and third-generation EGFR TKIs in this population have also been reported. A significant PFS and overall survival (OS) benefit has been demonstrated for osimertinib and dacomitinib [4–6] compared with gefitinib or erlotinib. The optimal treatment sequence for patients with EGFRm aNSCLC continues to evolve, related largely to an increasing number of breakthroughs and studies in this field.

Although the efficacy of EGFR-TKIs in the treatment of EGFRm aNSCLC patients is well established, the treatment decision-making process is becoming more complex as our knowledge of EGFRm and resistance pathways grows and more treatment options become available. Thus, treating physicians must consider an increasing number of factors, facing real world challenges. Indeed, a consensus developed by experts could help to guide decisions and issues that can occur in clinical practice in case there is not enough evidence.

We have establish a consensus about the decision criteria for selecting the best first-line (1L) therapy, the importance of a second biopsy on disease progression to determine the most appropriate subsequent therapies using the Delphi Technique method [7].

## Materials and methods

### Objectives

The aim of this study was to establish a consensus through Delphi Technique on the treatment strategy options in Spain for aNSCLC harboring EGFRm. An external expert in Delphi methodology guaranteed the quality of the overall process.

### Study design

#### Scientific Committee

In September 2019, a scientific committee of six experts in comprehensive lung cancer management met to define and lead this consensus project. The following steps were carried out by the scientific committee: (a) selection of expert panel; (b) generation of clinical statements based on review of the medical literature; (c) definition of the consensus levels and agreement on methodology; (d) interpretation of the results from the two Delphi rounds (DR); (e) develop of the final consensus document.

##### Expert panel

A panel of 31 medical oncologists specialized in lung cancer and outstanding for their clinical and research experience were selected by the scientific committee. In this selection of an expert panel, a balanced territorial representation of Spain was considered.

### Delphi

#### Generation of Statements

After a narrative literature review, 33 statements organized in 4 major domains were drafted by the scientific committee. These domains aimed to cover from the key diagnostic criteria to different treatment lines according to the patient profile:

1. Molecular diagnosis on aNSCLC (10 statements);
2. histologic profile and patient clinical characteristics (7 statements);
3. 1L therapy in EGFRm aNSCLC (8 statements);
4. second and subsequent-lines treatment in EGFRm aNSCLC (8 statements).

It was established that these statements would be evaluated in patients with EGFRm aNSCLC with good general condition (ECOG PS 0 and 1) and without medical contraindications to receive treatment. Only reimbursed treatments in Spain are considered.

### Consensus levels

The expert panel completed two DR of the statements through questionnaire in an online platform.

Each item was responded using Likert scale with 1–9 range (1 = “strongly disagree”; 9 = “strongly agree”) and the consensus level was calculated by tertiles. Range from 1 to 3 were considered values of disagreement, 4–6 range neither disagree nor agree, and scores from 7 to 9 were considered values of agreement. A statement with consensus existed when more than 2/3 of the answers are in the median range (MR), and not consensus if more than 33.3% are outside the MR. If the MR is in 7–9, there is consensus about agreement between panelists, if MR is between 4–6 exist indeterminate consensus and when the MR is 1–3 there is consensus of panelists disagreement.

The possible consensus levels were the following:

- Consensus/agree: there is consensus between panelist, the statement should not be reviewed.
- Consensus/disagree: there is consensus that panelists disagree.
- No consensus/agree: there are more panelists in agreement, but there is no consensus.
- No consensus/disagree: there are more answers in disagreement, but there is no consensus.
- Consensus/indeterminate: majority neither agree nor disagree. Statements should be reviewed and to decide whether to delete or keep up once evaluated (no items achieved this score).
- No consensus/indeterminate: there is no general agreement or disagreement, and, furthermore, there is no consensus on this indeterminacy.

All statements reaching consensus in the first round (FDR) did not undergo the second Delphi round (SDR). A SDR was held for the 8 statements without consensus in the FDR.

In FDR, started on 30 January 2020, 31 panelists answered the complete questionnaire, which supposes a response rate of 100%. In SDR (23 March 2020) 30 of 31 completed the questionnaire (96.8% response rate). This study consisted of a survey of expert opinions and no patient data were collected, so no specific independent ethical or research review or approval was necessary.

### Statistical analysis

The reliability of the questionnaire was assessed by internal consistency using Cronbach’s alpha coefficient (Cronbach’s *α*) and intraclass correlation (*r*_*i*_) coefficients. Both statistics were calculated for the complete questionnaire and for each domain.

Median and interquartile range (p25–p75) were calculated for each item (none of the variables showed Gaussian distribution). As it mentioned above, the score responses were divided in tertiles (ranges: 1–3, 4–6, 7–9). The agreement level was calculated according to the tertile in which the MR of the responses was located, and the consensus level was evaluated by the percentage of answers in this median tertile.

Spearman's correlation coefficient, following Martinez et al. criteria [8], was calculated to assess the correlation between two rounds for the total Delphi questionnaire as well as between domains and for the eight statements evaluated in both rounds. Further, the quantitative concordance was calculated by Kappa index (by Ashby [9].)

The SPSS 21.0. was used for the statistical analyses.

### Development of the consensus manuscript

This project began in September 2019 and ended in July 2020. During this time and when this article was drafted, newly published literature and congress presentations were reviewed to analyze the clinical implications of any new data in patients with aNSCLC and to provide support for the consensus statements.

## Results

In the FDR (100% response rate), 33 statements were evaluated, and a consensus was reached for 24 statements (72.7%). One of the non-consensus statements was rejected after reassessed by the scientific committee, and the remaining 8 statements were evaluated in the SDR (96.8% response rate), in which neither achieved consensus.

### Reliability of the questionnaire

Cronbach's alpha coefficient and intraclass correlation coefficients showed a high internal consistency for the complete questionnaire as well as for each domain with values above 0.7 in general (except *r*_*i*_ Domain 1 and 3) (Table 1).

**Table 1:** Evaluation of Delphi questionnaire consistency

	DR	Cronbach's *α* (*p*)^a^	*r*_*i*_ (*p*)^a^
TOTAL (33 ítems)	1	0.837 (< 0.001)	0.750 (< 0.001)
	2	0.834 (< 0.001)	0.746 (< 0.001)
Domain 1: molecular diagnosis on aNSCLC (10 items)	1	0.761 (< 0.001)	0.679 (0.006)
	2	0.751 (< 0.001)	0.668 (0.006)
Domain 2: histologic profile and patient clinical characteristics (7 items)	1	0.815 (< 0.001)	0.787 (< 0.001)
	2	0.829 (< 0.001)	0.808 (< 0.001)
Domain 3: first-line treatment in aNSCLC with EGFRm positive (8 items)	1	0.712 (< 0.001)	0.681 (< 0.001)
	2	0.712 (< 0.001)	0.681 (< 0.001)
Domain 4: second and subsequent-lines’ treatment in aNSCLC with EGFRm positive (8 items)	1	0.715 (< 0.001)	0.702 (< 0.001)
	2	0.718 (< 0.001)	0.712 (< 0.001)

*aNSCLC* Advanced non–small-cell lung cancer, *Cronbach's α* Cronbach's alpha coefficient, *DR* Delphi round, *EGFRm* epidermal growth factor receptor mutation, *r*_*i*_ intraclass correlation coefficient

^a^Acceptable values for research purposes are considered values above 0.7, between 0.7–0.9 high values of reliability and above 0.9 very high values

### Molecular diagnosis on aNSCLC

Table 2 shows the responses for the ten statements in domain 1 which is focused on the importance of molecular diagnosis prior to treatment election. Eight items reached a high panelist consensus (> 78%) about the agreement of these statements. Only one did not reach consensus neither in the FDR nor in the SDR. The other one statement was deleted from the SDR.

**Table 2:** Domain 1: molecular diagnosis on aNSCLC

Statement	DR	^a^MR	Panelist in MR, n(%)	Panelist in ^b^p1/p2 range, n(%)	Consensus
1. I always wait to have the staging of the disease before requesting the molecular diagnosis	1	4–6	7(22.6)	12(38.7)/12(38.7)	No consensus/indeterminate
	2	7–9	18(60.0)	5(16.7)/7(23.3)	No consensus/disagree
2. Prior to establish a treatment in non-smoking patients, the EGFR mutation status must be studied in metastatic or advanced NSCLC, regardless of histology	1	7–9	27(87.1)	3(9.7)/1(3.2)	Consensus/agree
	2	N/A	N/A	N/A	N/A
3. Prior to establish a treatment, the EGFR mutation status must be studied in entire aNSCLC	1	7–9	30(96.8)	0/1(3.2)	Consensus/agree
	2	N/A	N/A	N/A	N/A
4. Prior to establish an aNSCLC treatment, I always wait to definitive EGFR mutation status, regardless of the percentage of PD-L1/PD-L1 expression	1	7–9	31(100)	0/0	Consensus/agree
	2	N/A	N/A	N/A	N/A
5. Whether there is enough tissue sample I always try to use them for starting the EGFR mutation status analysis	1	7–9	30(96.8)	0/1(3.2)	Consensus/agree
	2	N/A	N/A	N/A	N/A
6. The EGFR mutation status analysis is essential. If there is not enough tissue sample, I consider taking a second sample	1	7–9	28(90.3)	3(9.7)/0	Consensus/agree
	2	N/A	N/A	N/A	N/A
7. If there is not enough tissue sample, a liquid biopsy is requested to determine the EGFR mutation status	1	7–9	30(96.8)	1(3.2)/0	Consensus/agree
	2	N/A	N/A	N/A	N/A
8. In case of result of EGFRm-negative in tissue sample, I always perform a confirmation test in liquid sample (plasma)	1	1–3	17(54.8)	11(35.5)/3(9.7)	No consensus/disagree
	2	N/A	N/A	N/A	N/A
9. For establishing a treatment to the patient, I always wait the results of specific biomarkers for targeted therapy (EGFR, ALK, ROS1)	1	7–9	30(96.8)	1(3.2)/0	Consensus/agree
	2	N/A	N/A	N/A	N/A
10. It is appropriated to use of NGS platform for identification of genetic-based biomarkers	1	7–9	30(96.8)	1(3.2)/0	Consensus/agree
	2	N/A	N/A	N/A	N/A

*aNSCLC* Advanced non–small-cell lung cancer, *DR* Delphi round, *EGFR* epidermal growth factor receptor mutation, *MR* median range, *NA* not applicable, *NGS* next-generation sequencing, *NSCLC* non–small-cell lung cancer

^a^MR = 7–9 agree range, MR = 4–6 neither agree nor disagree rage, MR = 1–3 disagree range

^b^p1/p2 ranges depend of MR: when MR = 1–3 range, *p*1 = 4–6 and *p*2 = 7–9; if MR = 4–6, *p*1 = 1–3 and *p*2 = 7–9; finally if MR = 7–9, *p*1 = 1–3 and *p*2 = 4–6

Highlight the unanimity of agreement between experts (100% consensus) about the need to know the EGFR mutation status, regardless of PD-L1/ PD-L1 expression, before to establish the treatment (statement 4).

The one statement with no consensus asked about the importance of disease stage versus molecular diagnosis (statement 1). On the other hand, the statements with high consensus were focused on EGFR status determination (preferability from tissue samples) and analysis of the different biomarkers, which shows that both variables are key and influence the treatment decision and strategy in patients with aNSCLC.

### Histologic profile and patient clinical characteristics

Table 3 shows the responses for the 7 statements in domain 2 that was focused on the relevance of histologic profile and patient clinical characteristics regarding the 1L therapy election. This was the domain with less consensus. The specialists only coincided with the no relevance of smoking habit in establishing the 1L therapy and the importance of always perform a CNS image at diagnosis.

**Table 3:** Domain 2: histologic profile and patient clinical characteristics

Statement	DR	^a^MR	Panelist in MR, n(%)	Panelist in^b^p1/p2 range, n(%)	Consensus
1. The age of the patient^c^ is key to establish the 1L therapy	1	1–3	19(61.3)	8(25.8)/4(12.9)	No consensus (limit)/disagree
	2	1–3	16(53.3)	5(16.7)/9(30.0)	No consensus/disagree
2. The smoking habit of the patient^c^ is key to establish the 1L therapy	1	1–3	24(77.4)	5(16.1)/2(6.5)	Consensus/disagree
	2	NA	NA	NA	NA
3. ECOG PS of the patient^c^ is key to establish the 1L therapy	1	4–6	9(29.0)	10(32.3)/12(38.7)	No consensus/indeterminate
	2	4–6	8(26.7)	11(36.7)/11(36.7)	No consensus/indeterminate
4. The comorbidities of the patient^c^ is key to establish the 1L therapy	1	4–6	6(19.4)	10(32.3)/15(48.4)	No consensus/indeterminate
	2	4–6	9(30.0)	10(33.3)/11(36.7)	No consensus/indeterminate
5. The common EGFR mutation subtypes (del19 and L858R) are key to establish a 1L therapy	1	4–6	7(22.6)	15(48.4)/9(29.0)	No consensus/indeterminate
	2	1–3	17(56.7)	5(16.7)/8(26.7)	No consensus/disagree
6. The presence of brain metastases in the patient^c^ is key to establishing the 1L therapy	1	7–9	18(58.1)	7(22.6)/6(19.4)	No consensus/agree
	2	7–9	19(63.3)	4(13.3)/7(23.3)	No consensus (limit)/agree
7. The patient^c^ always undergo to CNS imaging (MRI/CT) at diagnosis	1	7–9	29(93.5)	2(6.5)/0	Consensus/agree
	2	NA	NA	NA	NA

*CNS* Central nervous system, *CT* computed tomography, *DR* Delphi round, *EGFR* epidermal growth factor receptor mutation, *1L* first line, *MR* median range, *MRI* magnetic resonance imaging, *NA* not applicable

^a^MR = 7–9 agree range, MR = 4–6 neither agree nor disagree rage, MR = 1–3 disagree range

^b^*p*1/*p*2 ranges depend of MR: When MR = 1–3 range, *p*1 = 4–6 and *p*2 = 7–9; if MR = 4–6, *p*1 = 1–3 and *p*2 = 7–9; finally if MR = 7–9, *p*1 = 1–3 and *p*2 = 4–6

^c^patient with aNSCLC and EGFRm

### 1L therapy in patients with aNSCLC and EGFRm positive

Table 4 shows the responses for the eight statements in domain 3 which was focused on the therapeutic election as 1L therapy in EGFRm aNSCLC patients. Outcomes show there is homogeneous therapeutic criteria, with consensus in all the evaluated items (74.2–96.8%).

**Table 4:** Domain 3: First-line treatment in EGFRm advanced NSCLC

Statement	DR	^a^MR	Panelist in MR, *n*(%)	Panelist in ^b^p1/p2 range, *n*(%)	Consensus
1. The best therapeutic option is chosen as 1L therapy, regardless of the subsequent lines	1	7–9	29(93.5)	1(3.3)/1(3.2)	Consensus/agree
	2	NA	NA	NA	NA
2. The first-generation of EGFR-TKI (gefitinib, erlotinib) are the most effective and safe 1L therapy in patients with common activating mutations (del19, L858R)	1	1–3	25(80.6)	6(19.4)/0	Consensus/disagree
	2	NA	NA	NA	NA
3. The second-generation EGFR-TKI (afatinib) is the most effective and safe 1L therapy in patients with common activating mutations (del19, L858R)	1	1–3	23(74.2)	8(25.8)/0	Consensus/disagree
	2	NA	NA	NA	NA
4. The 3rd-generation EGFR-TKI (osimertinib) is the most effective and safe 1L therapy in patients with common activating mutations (del19, L858R)	1	7–9	30(96.8)	0/1(3.2)	Consensus/agree
	2	NA	NA	NA	NA
5. In case of the patient has uncommon EGFR mutations, the treatment choice is different	1	7–9	24(77.4)	5(16.1)/2(6.5)	Consensus/agree
	2	NA	NA	NA	NA
6. The benefit of osimertinib in progression-free survival compared to other EGFR-TKI is relevant and justifies its use as 1L therapy	1	7–9	29(93.5)	2(6.5)/0	Consensus/agree
	2	NA	NA	NA	NA
7. The benefit that osimertinib provides in overall survival compared to other EGFR-TKI is relevant and justifies its use as 1L therapy	1	7–9	29(93.5)	2(6.5)/0	Consensus/agree
	2	NA	NA	NA	NA
8. The safety profile of osimertinib is relevant to support its use as 1L therapy	1	7–9	29(93.5)	1(3.2)/1(3.2)	Consensus/agree
	2	NA	NA	NA	NA

*DR* Delphi round, *EGFR-TKI* epidermal growth factor receptor tyrosine kinase inhibitor, *1L* first line, *MR* median range, *NA* not applicable

^a^ MR = 7–9 agree range, MR = 4–6 neither agree nor disagree rage, MR = 1–3 disagree range

^b^*p*1/*p*2 ranges depend of MR: When MR = 1–3 range, *p*1 = 4–6 and *p*2 = 7–9; if MR = 4–6, *p*1 = 1–3 and *p*2 = 7–9; finally if MR = 7–9, *p*1 = 1–3 and *p*2 = 4–6

Osimertinib was considered the best 1L therapy choice in patients with common activating mutations (del19, L858R) due the safety profile, PFS and OS of this drug compared to other EGFR-TKI, and for patients with uncommon EGFR mutations.

### Second and subsequent-lines treatment in aNSCLC with EGFRm positive

Table 5 shows the responses for the eight statements in domain 4 which was focused on the second and subsequent-lines therapeutic options in patients with EGFRm aNSCLC. As in the front-line, the specialists showed homogeneous therapeutic criteria with consensus in 6/8 of the statements, highlighting the 100% consensus in three of them.

**Table 5:** Domain 4: Second and subsequent-lines treatment in EGFRm advanced NSCLC

Statement	DR	^a^MR	Panelist in MR, *n* (%)	Panelist in ^b^p1/p2 range, *n* (%)	Consensus
1. In case of NSCLC oligoprogression during 1L therapy with osimertinib, osimertinib is maintained plus local treatment	1	7–9	31(100)	0/0	Consensus/agree
	2	NA	NA	NA	NA
2. In case of progression to osimertinib treatment, PD-L1 expression is relevant for taking future decision	1	4–6	9(29.0)	12(38.7)/10(32.3)	No consensus/indeterminate
	2	1–3	15(50.0)	7(23.3)/8(25.8)	No consensus/disagree
3. In case of progression to osimertinib treatment, the priority is to include the patient in a clinical trial	1	7–9	30(96.8)	0/1(3.2)	Consensus/agree
	2	NA	NA	NA	NA
4. In case of progression to osimertinib treatment, to perform a new biopsy is relevant	1	7–9	28(90.3)	3(9.7)/0	Consensus/agree
	2	NA	NA	NA	NA
5. In case of progression to treatment with a first-generation TKi (gefitinib, erlotinib), to perform a new biopsy is relevant	1	7–9	31(100)	0/0	Consensus/agree
	2	NA	NA	NA	NA
6. After progression to treatment with a second generation TKi (afatinib), to perform a new biopsy is relevant	1	7–9	31(100)	0/0	Consensus/agree
	2	NA	NA	NA	NA
7. In case of progression to osimertinib or a TKi treatment in absence of T790M, the best option is to use chemotherapy	1	7–9	21(67.7)	7(22.6)/3(9.7)	Consensus (limit)/agree
	2	NA	NA	NA	NA
8. In case of progression to osimertinib or a treatment in absence of T790M mutation, the best option is to use chemotherapy + antiangiogenic therapy + anti-PD-L1 antibody	1	7–9	17(54.8)	11(35.5)/3(9.7)	No consensus/agree
	2	4–6	13(43.3)	3(10.0)/14(46.7)	No consensus/indeterminate

*DR* Delphi round, *1L* first line, *MR* median range, *NA* not applicable, *NSCLC* non–small-cell lung cancer, *PD-L1* programmed death-ligand 1, *TKI* tyrosine kinase inhibitor

^a^MR = 7–9 agree range, MR = 4–6 neither agree nor disagree rage, MR = 1–3 disagree range

^b^*p*1/*p*2 ranges depend of MR: When MR = 1–3 range, *p*1 = 4–6 and *p*2 = 7–9; if MR = 4–6, *p*1 = 1–3 and *p*2 = 7–9; finally if MR = 7–9, *p*1 = 1–3 and *p*2 = 4–6

In patients treated with osimertinib in front-line, if oligoprogression occured during the therapy, this TKI must be maintained adding local treatment. In case of progression, evaluation of PD-L1 expression was needed in order to assess properly the patient, and the inclusion in a clinical trial would be a priority. To perform a new biopsy was relevant in case of progression, regardless of the type of TKI used on the front-line. Finally, in case of disease progression to osimertinib or in absence of T790M mutation, the best option was to use chemotherapy.

### Correlation analysis

In order to assess the correlation between the two rounds as much the total questionnaire, as each domain (Table 6a) and of the eight items which were included in both rounds (Table 6b), Spearman's coefficient and Kappa index were calculated. The Kappa index values corroborate Spearmans`s coefficient estimators in all cases.

**Table 6:** Delphi Rounds correlation analysis

A. Correlation of the total questionnaire and of the domains between two Delphi Rounds		
	Spearman’s c (*p*)^a^	Kappa index (*p*)^b^
TOTAL (33 ítems)	0.909 (< 0.001)	0.880 (< 0.001)
Domain 1. Molecular diagnosis on aNSCLC (10 items)	0.954 (< 0.001)	0.939 (< 0.001)
Domain 2. Histologic profile and patient clinical characteristics (7 items)	0.856 (< 0.001)	0.829 (< 0.001)
Domain 3. First-line treatment in aNSCLC with EGFRm positive (8 items)	1 (< 0.001)	1 (< 0.001)
Domain 4. Second and subsequent-lines treatment in aNSCLC with EGFRm positive (8 items)	0.849 (< 0.001)	0.867 (< 0.001)

B. Correlation of the statements between the two Delphi Rounds		
Statement	Spearman’s c (*p*)^a^	Kappa index (*p*)^b^
Domain 1. Molecular diagnosis on aNSCLC		
Item 1	0.318 (0.125)	0.359 (0.137)
Domain 2. Histologic profile and patient clinical characteristics		
Item 1	0.892 (< 0.001)	0.860 (< 0.001)
Item 3	0.902 (< 0.001)	0.899 (< 0.001)
Item 4	0.738 (0.006)	0.708 (0.008)
Item 5	0.561 (0.162)	0.569 (0.156)
Item 6	0.856 (< 0.001)	0.842 (< 0.001)
Domain 3. First-line treatment in aNSCLC with EGFRm positive		
Domain 4. Second and subsequent-lines treatment in aNSCLC with EGFRm positive		
Item 2	0.662 (0.016)	0.641 (0.022)
Item 6	0.692 (0.019)	0.715 (0.011)

*aNSCLC* Advanced non–small-cell lung cancer, *EGFRm* epidermal growth factor receptor mutation, *Spearman's c* Spearman's correlation coefficient

^a^Cuantitative correlation score: [0—0.25] poor correlation; [0.26–0.50] weak correlation; [0.51–0.75- moderate/strong; [0.76–1] strong/very strong correlation

^b^Cualitative correlation score: < 0.00 no agreement; 0.00–0.20- poor concordance; 0.21–0.40- weak concordance; 0.41–0.60- moderate concordance; 0.61–0.80- good concordance; 0.81–1- very good concordance

Table 6a shows a strong quantitative correlation between two DR

(Spearmans’s coefficient range 0.76–1 = strong or very strong correlation) both in total questionnaire and by domains. However, the coefficients are overestimated because the values of the items which reaches consensus at the first round are repeated in the SDR. Kappa index indicates a good qualitative concordance in total questionnaire as well as in each domain.

The items between the two phases have moderate and strong correlation values, except for item 1 from Domain 1 with weak correlation (Table 6b).

Finally, the coefficient of variation was much less than 10%. Such a low value, which did not even reach 1%, supports the decision not to carry out a new round, since it would not provide variation.

## Discussion

The current Delphi study shows that a high degree of consensus exists among experts on the relevance to obtain molecular diagnosis before starting any treatment in aNSCLC, how these patients should be treated in the 1L setting according to the presence of EGFRm, and how the 1L therapy decision in these population may influence subsequent therapeutic approaches. This consensus document complements the information in the European Society of Medical Oncology (ESMO) [10], Sociedad Española de Oncología Médica (SEOM) and Sociedad Española de Anatomía Patológica (SEAP) [11], American College of Pathologists (ACP) [12] and aims to provide physicians with a specific therapeutic decision process to optimize management of EGFRm aNSCLC patients.

Regarding molecular diagnosis, it is important to highlight the unanimity of agreement among experts about the need to know the definitive EGFRm status, regardless of PD-L1 expression, or patient characteristics like age or smoking habit, before starting aNSCLC treatment. This recommendation follows the most relevant and current guidelines [10–12]. Although tumor tissue is the most relevant source of information, unfortunately in up to 25% of cases [13], the initial tissue biopsy is inadequate for precision oncology. In this context, the use of liquid biopsy is an efficient diagnostic alternative which can be considered at the time of initial diagnosis in patients who need tumor molecular profiling, specially when tumor tissue is scarce, unavailable, or a delay greater than 2 weeks in obtaining tumor tissue is expected [14, 15]. The one statement with no consensus was related to the importance of disease stage versus molecular diagnosis. After the ADAURA trial, that has shown significant results in terms of disease free survival with osimertinib in the adjuvant setting in EGFRm stage IB-IIIA population [16], probably changing the current treatment paradigm in early NSCLC EGFRm, all patients will need to be tested for EGFRm regardless of the stage of disease.

Moving forward the histologic profile and patient clinical characteristics, the experts agreed with the no relevance of smoking habit and age in establishing the 1L therapy and the importance of CNS imaging at diagnosis, given the high incidence of brain metastasis in EGFRm aNSCLC [17]. Regarding the type of EGFRm, the two most common EGFRm subtypes are a deletion in exon 19 (Del19) or point mutations in exon 21 (L858R) that constitute approximately 90% of activating mutations. Although subgroup analysis from several clinical trials have shown that a Del19 mutation is predictive of better efficacy than L858R mutation,the EGFRm subtype is not a factor to be considered in the EGFR-TKI selection process in this consensus [3]. On the contrary, patients with aNSCLC with uncommon EGFRm may experience variable responses to the currently available EGFR-TKIs [18, 19]. In patients with uncommon EGFRm, there was a consensus that the treatment of choice had to be different.

Regarding the 1L therapy option in EGFRm, osimertinib was considered the best front-line choice in patients with common activating mutations (del19, L858R). The FLAURA trial results support this recommendation: osimertinib demonstrated improvement in median PFS (18.9 months vs. 10.2 months; hazard ratio (HR) 0.46; 95% CI, 0.37 to 0.57; *p* < 0.001) and a more favorable toxicity profile due to its lower affinity for wild-type EGFR. Furthermore, osimertinib has also improved efficacy against brain metastases [4]. Very recently published OS results have shown a median of 38.6 months in the osimertinib group and 31.8 months in the comparator group (HR, 0.80; 95.05% CI, 0.64 to 1.00; *P* = 0.046). At 3 years, 79 of 279 patients (28%) in the osimertinib group and 26 of 277 (9%) in the comparator group were continuing to receive a trial regimen [5]. Grade ≥ 3 adverse events were reported in 42% of the patients in the osimertinib group and in 47% of those in the comparator group. These data favor osimertinib for tolerability and safety, except for the slightly higher rate of interstitial lung disease, but which was nonetheless manageable [20].

Focusing on the second and subsequent treatment lines in EGFRm aNSCLC there was a consensus to maintain TKI plus local treatment in case of NSCLC oligoprogression during 1L therapy. This statement has been validated in small trials showing the benefit of this strategy specially with the use of sterotactic body radiation therapy (SBRT) [21, 22]. In case of progression to osimertinib, the inclusion in a clinical trial and to perform a new biopsy was considered to be relevant. To perfom a new biopsy was also considered to be relevant in case of progression to first or second generation TKI in order to determine the acquired resistance mechanism. Finally, in case of disease progression to osimertinib or after first or second generation TKI in absence of T790M mutation, the best option was to use chemotherapy.

Although this consensus document aims to help therapeutic decision-making process, it has several limitations. First, this Delphi project has been developed under the premise of patients with EGFRm aNSCLC with a good general condition (ECOG PS 0 and 1) and without medical contraindications. This may limit its potential applicability to all patients with EGFRm aNSCLC. Also, as the treatment of aNSCLC further evolves, it is important to underline that the SDR took place before the communication of some relevant scientific information such as the results of osimertininb in adjuvant setting in stage IB-IIIA NSCLC [16].

Despite these limitations, the study shows the consistency of the questionnaire created to meet the proposed objectives statements selected: Cronbach's alpha coefficient and intraclass correlation coefficients showed a high internal consistency for the complete questionnaire as well as for each domain with values. The correlation analysis showed strong quantitative correlation and a good qualitative concordance between two DR in total questionnaire as well as in each domain. The Kappa index values corroborate Spearmans’s coefficient estimators in all cases.

This consensus document has been generated due to the need to answer key clinical questions such as the best sequencing approach for EGFRm aNSCLC. We would encourage to use this document to help stimulate discussion on future real-world studies that could be carried out to support or question the consensus statements.
